# Quality antenatal care services delivery at health facilities of Ethiopia, assessment of the structure/input of care setting

**DOI:** 10.1186/s12913-020-05372-6

**Published:** 2020-06-01

**Authors:** Atkure Defar, Theodros Getachew, Girum Taye, Tefera Tadele, Misrak Getnet, Tigist Shumet, Gebeyaw Molla, Geremew Gonfa, Habtamu Teklie, Ambaye Tadesse, Abebe Bekele

**Affiliations:** 1 Reproductive Health Research Team, Ethiopian Public Health Institute, Addis Ababa, Ethiopia; 2 Department of Epidemiology and Biostatistics, University of Gondar, College of Medicine and Health Science, Institute of Public health, Gondar, Ethiopia; 3 Health System Research Team, Ethiopian Public Health Institute, Addis Ababa, Ethiopia; 4 College of Medicine and Health Science, Institute of Public health, University of Gondar, Gondar, Ethiopia; 5 Health System and Reproductive Health Research Directorate, Ethiopian Public Health Institute, Addis Ababa, Ethiopia

**Keywords:** Antenatal care, Ethiopia, Quality, Structure, Input, Readiness score

## Abstract

**Background:**

According to the Donabedian model, the assessment for the quality of care includes three dimensions. These are structure, process, and outcome. Therefore, the present study aimed at assessing the structural quality of Antenatal care (ANC) service provision in Ethiopian health facilities.

**Methods:**

Data were obtained from the 2018 Ethiopian Service Availability and Readiness Assessment (SARA) survey. The SARA was a cross-sectional facility-based assessment conducted to capture health facility service availability and readiness in Ethiopia. A total of 764 health facilities were sampled in the 9 regions and 2 city administrations of the country. The availability of equipment, supplies, medicine, health worker’s training and availability of guidelines were assessed. Data were collected from October–December 2017. We run a multiple linear regression model to identify predictors of health facility readiness for Antenatal care service. The level of significance was determined at a *p*-value < 0.05.

**Result:**

Among the selected health facilities, 80.5% of them offered Antenatal care service. However, the availability of specific services was very low. The availability of tetanus toxoid vaccination, folic acid, iron supplementation, and monitoring of hypertension disorder was, 67.7, 65.6, 68.6, and 75.1%, respectively. The overall mean availability among the ten tracer items that are necessary to provide quality Antenatal care services was 50%. In the multiple linear regression model, health centers, health posts and clinics scored lower Antenatal care service readiness compared to hospitals. The overall readiness index score was lower for private health facilities (β = − 0.047, 95% CI: (− 0.1, − 0.004). The readiness score had no association with the facility settings (Urban/Rural) (*p*-value > 0.05). Facilities in six regions except Dire Dawa had (β = 0.067, 95% CI: (0.004, 0.129) lower readiness score than facilities in Tigray region (*p*-value < 0.015).

**Conclusion:**

This analysis provides evidence of the gaps in structural readiness of health facilities to provide quality Antenatal care services. Key and essential supplies for quality Antenatal care service provision were missed in many of the health facilities. Guaranteeing properly equipped and staffed facilities shall be a target to improve the quality of Antenatal care services provision.

## Background

In a focused ANC program, four visits are required to guarantee the safety of a woman and her newborn through the provision of essential interventions [1]. However, in a revised World Health Organization guideline a minimum of eight ANC contacts is recommended in the absence of complications [2]. Therefore, to respond this need, health facilities should be well prepared in terms of trained human power, drugs, supplies, equipment and infrastructure.

Antenatal care is also an opportunity to promote the use of skilled attendance at birth and healthy behaviours such as breastfeeding, early postnatal care and planning for optimal pregnancy spacing [3]. Different community and facility-based studies have proved the negative effect of low Antenatal care service utilization and readiness of health facilities on the continuum of care, which flagged for a comprehensive program and research conduct [4].

Overall, to assess the service provision of Antenatal care services, four key components are required to be measured. These are; iron supplementation, tetanus toxoid vaccination, folic acid supplementation and monitoring for pregnancy-related hypertension disorder [5]. However, management of the Obstetric complications (such as preeclampsia, eclampsia, haemorrhage, tetanus toxoid immunization, and intermittent preventive treatment for malaria during pregnancy (IPTp) are among the essential intervention services that are required to be available at health facilities. The provision of these services should also be extended to the identification and management of infections [6].

However, it has shown negative improvement overtime in recent years with disparities by regions and facility types [7]. The recent Ethiopian Emergency Obstetric and Newborn Care assessment revealed that majority of health care facilities offered Antenatal service and not varied by regions, health facility type and ownership [8].

There were very few studies that assessed the quality of care for Antenatal care services [9–11]. These studies included few health facilities in limited geographic areas, focused on perceived quality of care and they assessed only factors associated with client perspective but not at facility level.

The availability and readiness of services are important factors that determine the health care service utilization [12]. However, it can not substitute service quality. Thus, measuring what is being delivered, rather than focusing on how often care is delivered is more important [10, 13].

Different models help to measure the quality of care provision [14]. Of these, the current study used Donabedian quality of care framework to measure structural quality. This study was the first of its kind to assess quality of Antenatal care service at the national level using structural indices. Therefore, this study aimed to assess the service availability, structural readiness of facilities and associated factors to provide quality Antenatal care service.

## Method

### Study area and data source

**Fig. 1:**
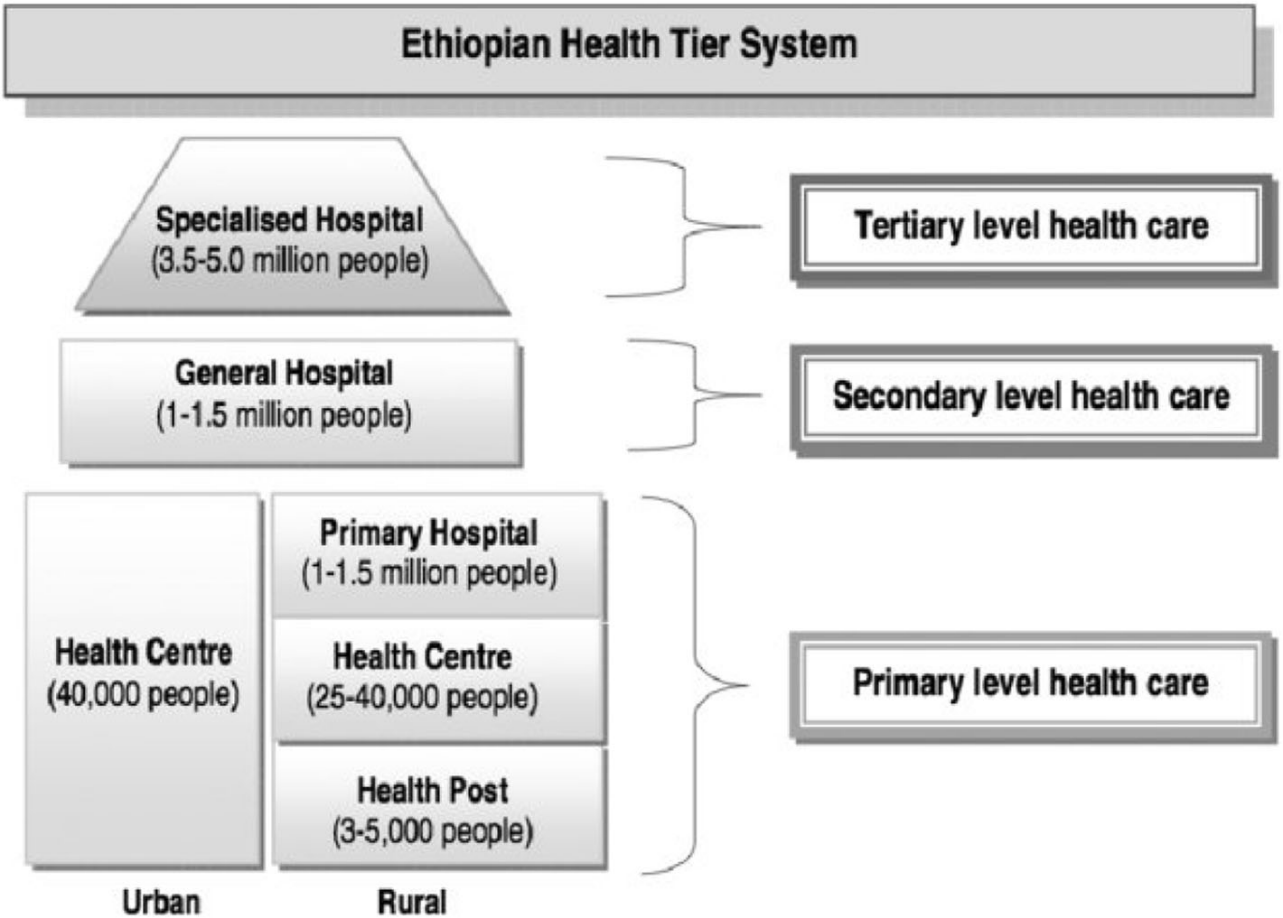
Ethiopian Health Tiers and Referral Structure (FMOH, HSTP 2015)

The Ethiopian health system has three tiers of service having more than 24,000 health facilities of different types [15]. The majority of services are provided at the primary level, which is organized around one primary hospital serving four or five Primary Health Care Units (PHCUs). As indicated in Fig. 1, a PHCU comprises a Primary hospital, health center and five satellite health posts. These serve as an approximate of 100, 000, 25,000 and 5000 people, respectively. A primary hospital is a referral center for the catchment area of the health center. General hospitals are the second level in health care tier system. These hospitals are referral centers for primary hospitals and provide service of approximately one million people. The tertiary level is specialized hospitals that serves about 5 million people [16].

The Ethiopian health service is augmented by the rapid expansion of the private for-profit and Non-Governmental Organization’s facilities that play a significant role in the health care system. They are concentrated largely in urban areas. However, the public sector delivers most of the health services in the country. This enhanced the Public-Private/NGO partnership and supported the health care services provided in the country.

Ethiopian has conducted Service Availability and Rreadiness Assessemnt in 2016 for the first time and recently in 2018. The present study used data from the 2018 SARA survey. The data was collected at a national level in all the nine regional states and two city administration (namely; Tigray, Afar, Amhara, Oromia, Somali, Benishangul-Gumuz, SNNP, Gambella, Harari, and two city administrations; i.e. Addis Ababa and Dire Dawa). The study was conducted from October to December 2017.

### Study design and sampling methods

A cross-sectional study design was conducted among selected health facilities in Ethiopia. The sample allocation considered the skewed regional level distribution of Ethiopian health facility. We used a stratified sampling technique. The stratification was based on facility type and managing authority. All hospitals, selected health centers, clinics and health posts were included in the sample. All health facilities that were included in the 2016 SARA were part of the 2018 SARA study [7] and included in the current analysis. New hospitals that were constructed afterwards in 2016 were included in the 2018 SARA survey.

### Sample size

The sample size was estimated using the following formula; *n* = [[(z2* p * q) + ME2] / [ME2+ z2* p * q / N]] *d,

Where *n* = the sample size, z = the square of the normal deviate at the required confidence level (3.84 is the square of the normal deviate (1.96) needed to provide an estimate at the 95% level of confidence) *p* = the proportion of facilities with the attribute of interest (A 47% of the Proportion of facilities with basic amenities from the 2016 SARA result was used), q = 1-p, ME = margin of error (15%), d = the design effect (because of the regional stratification we assumed a design effect of 1.5). *N* = Total number of Facilities in each stratum. Basic Amenities was defined as the mean availability of seven basic amenities, expressed in percentage. The items were; Electric power, Improved water source, Room with privacy, Adequate sanitation facilities, Communication equipment, Access to computer with internet, and Emergency transportation. The sample size was required to provide a national representation within 95% confidence level and +/− 15% precision. The sampling considered 20% for private clinics and additional 10% for refusals or closed facilities.

Thus, a total of 764 facilities were included in the study; 164 health centers, 165 clinics (Private facilities; either lower, medium or higher clinics) and 132 health posts. This number was within the range of 500 to 800 facilities of the World Health Organization recommendations for Service Availability and Readiness Assessment surveys that is required to have regional estimates (Table 1).

**Table 1:** Distribution of the study facilities by region, facility type and managing authority

Characteristics	Hospital	Health center	Health Post	Clinics	Total
Region	n(%)	n(%)	n(%)	n(%)	n(%)
Tigray	39(12.9)	14(8.5)	11(8.3)	12(7.3)	76(9.9)
Afar	6(2.0)	16(9.8)	12(9.1)	15(9.1)	49(6.4)
Amhara	65(21.5)	17(10.4)	16(12.1)	16(9.7)	114(14.9)
Oromia	75(24.8)	16(9.8)	16(12.1)	18(10.9)	125(16.4)
Somali	11(3.6)	16(9.8)	13(9.8)	17(10.3)	57(7.5)
Benishangul Gumuz	2(0.7)	15(9.1)	14(10.6)	14(8.5)	45(5.9)
S.N.N.P	58(19.1)	16(9.8)	16(12.1)	15(9.1)	105(13.7)
Gambella	3(1.0)	13(7.9)	12 (9.1)	14(8.5)	42(5.5)
Harari	5(1.7)	8(4.9)	11 (8.3)	11(6.7)	35(4.6)
Addis Ababa	32(10.6)	22(13.4)	0 (0.0)	23(13.9)	77(10.1)
Dire Dawa	7(2.3)	11(6.7)	11(8.3)	10(6.1)	39(5.1)
Managing Authority					
Public	243(80.2)	160(97.6)	132(100)	7(4.2)	542(70.9)
Others	60(19.8)	4(2.4)	0(0)	158(95.8)	222(29.1)
Location					
Urban	273(90.1)	86(52.4)	10(7.6)	135(81.8)	504(66.0)
Rural	30(9.9)	78(47.6)	122(92.4)	30(18.2)	260(34.0)
Total	303	164	132	165	764

### Data collection tool

The survey used computer-based electronic data collection using the Census and Survey Processing System (CSPro) data entry entry template. The data collection instruments were adopted from the WHO annual health facility monitoring guide [17]. Information on the health facility infrastructure; facility audit questions focused on the availability of trained personnel, clinical and laboratory services, medicines, and guidelines, information of location, and functional status components of support systems were collected. The measurement of the service availability and readiness of the ANC was made on four main components. These were; Iron Supplementation, Folic Acid supplementation, Tetanus Toxoid vaccination and Monitoring for the hypertensive disorder of pregnancy.

### Outcome variables

We measured the structural quality of care for Antenatal care service. To measure this, we used a combination of 10 trace items that are necessary to provide quality antenatal care service. These were availability of trained personnel in the past 2-years, ANC checklist and/or job aid, Guideline on ANC service, and Availability of supplies and drugs (availability of Hemoglobin, Urine dipstick, Iron Tablets, Folic Acid, Tetanus toxoid Vaccine, Intermittent Preventive Treatment in pregnancy, and Insecticide Treated bed Net. As a secondary outcome, we measured the overall service availability of ANC and components of antennal care service. Such as; Iron supplementation, Folic acid supplementation, Tetanus toxoid vaccination, and Monitoring for hypertensive disorder of pregnancy.

### Explanatory variables

Explanatory variables were; region, facility type, managing authority and facility setting (Urban/Rural).

### Data management and analysis

We used the WHO reference manual to select the structural readiness score items [17]. The items were counted for each case and added up to one number to calculate the mean. This gave us a continuous variable ranging from 0 to 10 as an outcome of interest. 

We used multiple linear regression model to determine the association between the overall index for quality of care and region, facility type, ownership, and facility setting. The SPSS version 20 and STATA version 14 software’s were used to do the analysis and ArcGIS version 10.4 was used to produce the map.

## Results

### Antenatal care service availability and readiness

The availability of ANC service was assessed using four components of the service. These were; Iron supplementation, folic acid supplementation, tetanus toxoid vaccination and monitoring for the hypertensive disorder of pregnancy. Antenatal care service was not offered in almost one in five of the surveyed facilities. Iron supplementation was available in 68.6% of the surveyed health facilities followed by Tetanus toxoid vaccination, that was 67.7%. Three-fourth and nearly two-thirds of surveyed facilities have provided monitoring for the hypertensive disorder of pregnancy and folic acid supplementation, respectively.

There was regional variation in offering ANC services. Higher than 89% of facilities in Tigray and Amhara regions provided ANC service, while the lowest (57.1%) was in Gambella. Antenatal care services were universally offered in general hospitals, primary hospitals and health centers. Whereas, only 28 and 29% of medium and lower clinics offered the service, respectively. In addition, tetanus toxoid vaccination offered in only 7 and 4% of the medium and lower clinics, respectively.

**Fig. 2:**
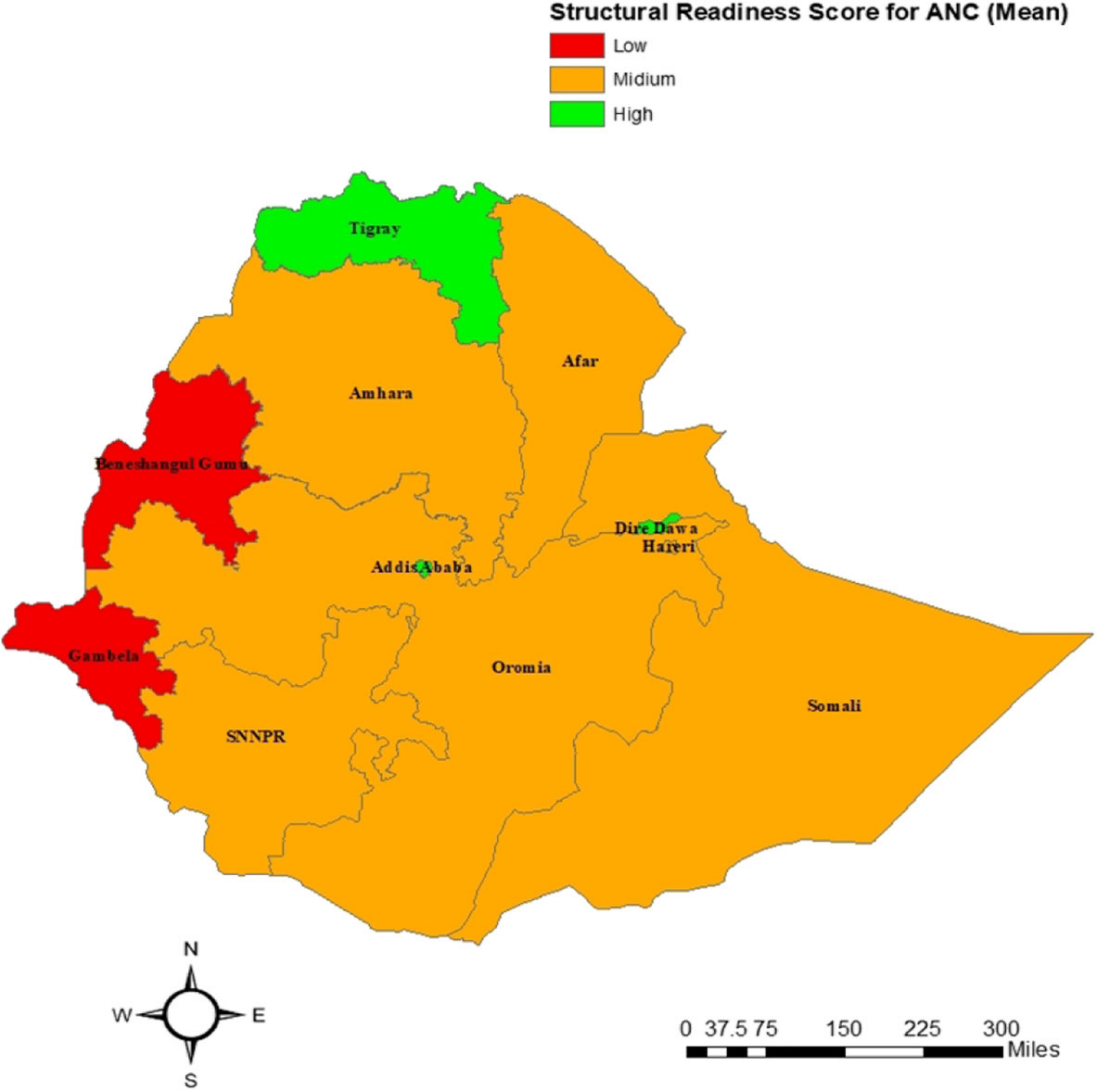
Maps showing the structural readiness score for Antenatal care by region in Ethiopia. (The authors produced the map and acknowledge the software (ArcGIS) used in the data management and analysis section)

Ninety-three percent of the government-owned facilities provided ANC service. The availability of ANC service was almost similar in both rural and urban health facilities. Seventy-eight percent of the facilities in Tigray and Afar offered iron supplementation, whereas the provision of monitoring of the hypertension disorder for pregnancy was highest in Tigray’s health facilities compared with other regions (Table 2 and Fig. 2).

**Table 2:** Percentage of facilities with ANC care and service by, regions, facility type, setting, *n* = 764

Facility Characteristics	Offers antenatal care		Supplementation				Tetanus toxoid vaccination		Monitoring for hypertensive disorder of pregnancy		Total number of facilities (^**a**^UW)
			Iron		Folic acid						
	n	%	n	%	N	%	n	%	n	%	n
**Region**											
Tigray	69	90.8	59	77.6	58	76.3	55	72.4	65	85.5	76
Afar	38	77.6	38	77.6	35	71.4	31	63.3	37	75.5	49
Amhara	101	88.6	86	75.4	84	73.7	79	69.3	94	82.5	114
Oromia	106	84.8	92	73.6	86	68.8	90	72.0	96	76.8	125
Somali	39	68.4	34	59.6	34	59.6	33	57.9	35	61.4	57
Benishangul -Gumuz	35	77.8	26	57.8	27	60.0	29	64.4	30	66.7	45
S.N.N.P	91	86.7	77	73.3	66	62.9	83	79.0	86	81.9	105
Gambella	24	57.1	19	45.2	18	42.9	21	50.0	22	52.4	42
Harari	27	77.1	24	68.6	24	68.6	22	62.9	26	74.3	35
Addis Ababa	56	72.7	47	61.0	48	62.3	48	62.3	54	70.1	77
Dire Dawa	29	74.4	22	56.4	21	53.8	26	66.7	29	74.4	39
**Facility type**											
Referral Hospital	29	93.5	27	87.1	27	87.1	28	90.3	29	93.5	31
General Hospital	116	100	103	88.8	100	86.2	96	82.8	115	99.1	116
Primary Hospital	154	98.7	139	89.1	132	84.6	134	85.9	152	97.4	156
Health Center	162	98.8	141	86.0	136	82.9	158	96.3	157	95.7	164
Health Post	104	78.8	84	63.6	75	56.8	87	65.9	74	56.1	132
Higher Clinic	8	42.1	6	31.6	6	31.6	6	31.6	8	42.1	19
Medium Clinic	21	28.4	14	18.9	14	18.9	5	6.8	19	25.7	74
Lower Clinic	21	29.2	10	13.9	11	15.3	3	4.2	20	27.8	72
**Managing Authority**											
Public	503	92.8	438	80.8	417	76.9	460	84.9	465	85.8	542
Others	112	50.5	86	38.7	84	37.8	57	25.7	109	49.1	222
**Facility setting**											
Urban	404	80.2	341	67.7	333	66.1	333	66.1	395	78.4	504
Rural	211	81.2	183	70.4	168	64.6	184	70.8	179	68.8	260
**Total number of facilities**^**a**^	**615**	**80.5**	**524**	**68.6**	**501**	**65.6**	**517**	**67.7**	**574**	**75.1**	**764**

^**a**^*UW* unweighted

Table 3 depicted the distribution of tracer items among facilities that offered ANC service. Accordingly, the mean availability of tracer items was highest in hospitals and health centers than other facility types. On average, clinics and health posts had three tracer items out of ten that are necessary to provide ANC service.

**Table 3:** Distribution of tracer items by facility type, *n* = 615

Tracer items measuring structural quality		Hospital		Health center		Health Post		Clinics		Total
		n	%	n	%	n	%	n	%	n
Guidelines available for antenatal care service		137	45.8	76	46.9	29	27.9	11	22.0	253
ANC check-lists and/or job-aids		235	78.6	132	81.5	52	50.0	23	46.0	442
At least one trained staff for antenatal care service		120	40.1	57	35.2	36	34.6	14	28.0	227
Blood pressure apparatus		295	98.7	156	96.3	54	51.9	49	98.0	554
Haemoglobin test		170	56.9	53	32.7	0	0	19	38.0	242
Urine dipstick protein test		287	96.0	111	68.5	0	0	27	54.0	425
Iron tablets		244	81.6	115	71.0	51	49.0	15	30.0	425
Folic acid tablets		251	83.9	117	72.2	49	47.1	13	26.0	430
Tetanus toxoid vaccine		205	68.6	119	73.5	32	30.8	6	12.0	362
ITNs		41	13.7	48	29.6	29	27.9	0	0.0	118
**Tracer item score**	**Mean**	**6.6**		**6.1**		**3.2**		**3.5**		**5.7**
	**SE**	**0.1**		**0.1**		**0.2**		**0.3**		**0.1**
**Total facilities (Unweighted)**		**299**		**162**		**104**		**50**		**615**

The availability of tracer items for the provision of ANC service in the hospital ranged from the highest, 98.7% for blood pressure apparatus and lowest to 14% for Insecticide Treated Nets (ITNs). Blood pressure apparatus was the most available (96.3%) tracer item in health centers followed by ANC check-lists and/or job-aids (81.5%). ITNs was the least available (29.5%) tracer item in health centers. However, the availability was higher among the health center than any other type of health facilities. Fifty-two percent the health post had Blood pressure apparatus, followed by checklists and/or job-aids (50%) and Iron tablets (49%), Blood pressure apparatus was the most available ANC service-related item in clinics (98%) while the least was ITNs, that was null. There were no kits for Haemoglobin and urine dipstick test in health posts **(**Table 3).

### Determinants of structural quality of ANC services

A linear regression model was used to check the association between facility type, managing authority, setting, and region with the readiness score to provide quality Antenatal care services. Accordingly, at 5% level of significance, health centers had (β = − 0.083, 95%CI: − 0.119, − 0.047), health posts (β = − 0.383, 95% CI: − 0.427, − 0.338), and clinics (β = − 0.389, 95%CI: − 0.434, − 0.344) lower readiness score compared with hospitals. The overall structural readiness score for quality ANC was lower for private health facilities (β = − 0.047, 95%CI: (− 0.1, − 0.004) than that of public health facilities and did not association with the locations of facilities (*p*-value > 0.05) (Table 4).

**Table 4:** Factors associated with structural readiness score of ANC service provision

Facility characteristics	ANC Readiness Score index	SE	Crude Model		Adjusted Model	
Facility type			B	95% CI	B	95% CI
Hospital *(Ref)*	6.6	.660				
Health center	6.1	.017	**−.056**	**(−.089, −.023)**	**−.083**	**(−.119, −.047)**
Health post	3.2	.018	**−.368**	**(−.403, −.332)**	**−.383**	**(−.427, −.338)**
Clinics^a^	3.5	.017	**−.398**	**(−.431, −.365)**	**−.389**	**(−.434, −.344)**
**Managing authority**						
Public *(Ref)*	5.8	.019	**.01**			
Others	5.1	.552	**−.019**	**(−.221, −.147)**	**−.047**	**(−.1 -.004)**
**Facility setting**						
Urban *(Ref)*	6.2	.011				
Rural	4.7	.018	**−.136**	**(−.172, −.100)**	−.026	(−.058, .006)
**Region**						
Tigray *(Ref)*	6.7	.028				
Afar	5.3	.044	**−.154**	**(−.241, −.068)**	−.039	(−.097, .019)
Amhara	5.6	.036	**−.119**	**(−.189, −.049)**	**−.132**	**(−.179, −.085)**
Oromiya	5.5	.035	**−.136**	**(−.204, −.067)**	**−.152**	**(−.198, −.106)**
Somali	5.8	.042	**−.129**	**(−.212, −.046)**	−.029	(−.085, .026)
Benishangul Gumuz	4.2	.045	**−.263**	**(−.352, −.174)**	**−.117**	**(−.178, −.057)**
S.N.N.P.	5.2	.036	−.156	**(−.227, −.085)**	**−.161**	**(−.209, −.115)**
Gambela	4.2	.046	−.297	**(−.387, −.205)**	**−.150**	**(−.212, −.089)**
Harari	5.9	.049	−.119	**(−.215, −.023)**	.019	(−.047, .084)
Addis Ababa	6.6	.039	−.083	**(−.160, −.007)**	**−.066**	**(−.12, −.013)**
Dire Dawa	6.5	.047	−.04	**(−.134, .053)**	.067	(.004, .129)

^a^Clinic: include higher, medium and lower clinics

There was regional difference in structural readiness to provide quality ANC services. Facilities in Amhara (β = − 0.132, 95%CI: (− 0.179, − 0.085)), Oromia (β = − 0.152, 95%CI: (− 0.198, − 0.106), Benishangul Gumuz (β = −.132, 95%CI: (− 0.178, − 0.057), SNNP. (β = − 0.161, 95%CI: (− 0.209, − 0.115), Gambella (β = − 0.15, 95%CI: (− 0.212, − 0.089), Addis Ababa (β = − 0.066, 95%CI: (− 0.12, − 0.013) regions had lower overall readiness score for ANC service than facilities in the Tigray region (*p*-value < 0.05). However, the overall ANC service readiness score for Dire Dawa city (β = 0.067, 95%CI: (0.004, 0.129) was higher than that of the Tigray region. The variables included in the model explained 60% of the variability (*R*^2^ = 0.6).

## Discussion

The availability of Antenatal care service was assessed using four components of the service. More than eight out of ten surveyed health facilities offered ANC service. The availability of the four components of ANC services was ranging from 65 to 75%. In overall, the mean availability of the ten tracer items that are necessary to provide quality ANC service was less than six. Hospitals were by far better in the provision of quality ANC service than other health facility types. Health centers, health posts and clinics had lower overall readiness score than hospitals. The overall readiness index score for private health facilities was lower than the public and had no difference by location of facilities. There was regional variation in readiness score.

In contrary to the current study, a study that used data from 20 nationally representative health facility assessments of both the SPA and the SARA revealed a higher proportion of health facility readiness to deliver iron supplementation (84.9%), tetanus toxoid vaccination (82.8%) and low result for hypertensive disease case management (23.0%) [18].

Our study demonstrated that there was a critical gap (40%) of health care providers who have been trained to provide ANC service. Congruently, the study conducted in Ugandan has shown an overall staffing gap of over 40% [19]. As the availability of trained health care providers is one of the strategies to improve the availability and quality of the service being provided, there should be a mechanism to overcome shortage of trained staff.

One of the determinant factors for the provision of quality of antenatal care service is the availability of equipment. In this regard, the current study has discovered that Ethiopian health facilities were poorly equipped. Similarly, a study conducted in Nigeria and Uganda showed as the quality of care was determined by the availability of medical equipment [19, 20]. If the measurement on the availability of equipment included the working status or the functionality, the result could have been different. Thus, to get the true picture of the quality of the service provided, assessments should consider the working status of the equipment.

A study conducted by Taddese and his collogues on quality of antenatal care services at public health facilities of Northwest of Ethiopia described that there was a lack of diagnostic reagents which was partly explained the problems observed in the provision of recommended antenatal care [21]. Similarly, in this study, the availability of diagnostic service for ANC was low and affected the overall readiness score.

The current study depicted the regional variation on service availability for ANC. Similarly, a multi-country study that used data from the Demographic and Health Survey has revealed the existence of geographic variation of ANC service readiness [22]. However, we did not observe any significant difference by facilities settings (Urban/Rural).

The overall ANC readiness score was fonnd to be low, which was 5.7. However, a study conducted in india had measured quality of ANC care in different ways using the clinical quality, interpersonal, quality of care and utilization of antennal care measures, likewise, It has found low quality of Antenatal care in the surveyed health facilities [23]. Contradicting to this, a study conducted to assess the quality of antenatal and childbirth care in selected rural health facilities in Burkina Faso, Ghana and Tanzania revealed a satisfactory level quality of Antenatal care service [24]. The study used the availability of infrastructure, essential equipment, essential drugs, vaccines and laboratory supplies as a measure of quality. However, to measure the structural readiness we used a WHO guideline for the assessment of health service availability and readiness. We have found that public health facilities were better in readiness to provide ANC service. Congruent with our findings, a study conducted in Vietnam and India has found that public health facilities were superior in readiness to that of the private health facilities [25, 26]. Moreover, our study finding was evinced by a multi-country study that was conducted in 46 Low and Middle-Income Countries [22]. The study reported that public health facilities were the main source of ANC services.

Findings from this study should be considered in the context of some limitations. Failure to compare and associate with many studies conducted in Ethiopia was one of the weaknesses of this study. This was due to the type and size of the data used. Besides, this study did not capture provider-level data that would tell more about the quality of care from the provider’s perspective. Thus, using a qualitative study is more helpful to understand the quality of care in the perspective of health care provider’s. The study has several strengths. We did the analysis using nationally representative data and we were able to generate evidence at regional level to show the geographic variability, which indirectly inform the service equity. In addition, unlike most available studies, this study focuses on showing facility-level structural quality.

## Conclusion

The overall readiness score to provide ANC service was low in Ethiopian health facilities. Majority of the health facilities lacks the key components of Antenatal care service. There was a difference in overall readiness of ANC service provision by region, facility type and ownership of the facility. In general, the availability and readiness of ANC service at health posts and clinics were below the expected. Addressing the regional disparity and provision of training for health care providers were found to be vital to improve the quality of service provision. As the quality and equity of maternal care are the core agenda of the health sector, attention shall be given to improve ANC service provision through proper resource allocation. Ensuring the availability of trained health workers, equipment, drugs and guidelines for Antennal care are the cornerstones for continuous improvement of facility-based health care services. Policymakers, program personnel, and partners shall use this evidence for the improvement of health service delivery. Further program evaluations and implementation researches are highly recommended to understand the multi-dimensional nature of the quality of care.

## Data Availability

The datasets used and/or analyzed during the current study are available in the data repository at the date center of the EPHI. It is also available with the corresponding author on a reasonable request.

## Abbreviations

CSPro: Census and Survey Processing System
IRB: Institutional Review Board
ITNs: Insecticides Treated Nets
LMICs: Low and Middle-Income Countries
IPT: Intermittent Preventive Treatment for Pregnancy
SARA: Service Availably and Readiness Assessment
ESPA+: Ethiopian Service Provision Assessment plus
